# Juvenile Spondyloarthropathies: Diagnostic and Therapeutic Advances—A Narrative Review

**DOI:** 10.3390/jcm14093166

**Published:** 2025-05-03

**Authors:** Călin Lazăr, Mirela Crișan, Oana-Iulia Man, Lucia Maria Sur, Gabriel Samașca, Alexandru Cristian Bolunduț

**Affiliations:** 1 1st Paediatric Clinic, Emergency Clinical Hospital for Children, 400370 Cluj-Napoca, Romania; calin.lazar@umfcluj.ro (C.L.); mirelacrisan_cluj@yahoo.co.uk (M.C.); oannaaman@yahoo.com (O.-I.M.); sur.maria@umfcluj.ro (L.M.S.); 2 1st Department of Paediatrics, “Iuliu Hațieganu” University of Medicine and Pharmacy, 400370 Cluj-Napoca, Romania; alexandru.bolundut@umfcluj.ro; 3 Department of Immunology, “Iuliu Hațieganu” University of Medicine and Pharmacy, 400162 Cluj-Napoca, Romania

**Keywords:** juvenile spondyloarthritis, enthesitis-related arthritis, biological treatment

## Abstract

Spondyloarthropathies (SpAs) represent a diverse group of seronegative immune-mediated inflammatory diseases characterized by a genetic predisposition and an association with human leukocyte antigen-B27. This narrative review aims to explore juvenile spondyloarthropathies (JSpAs), their classification, clinical manifestations, diagnostic challenges, and contemporary treatment strategies. According to the International League of Associations for Rheumatology criteria, JSpAs include several specific forms: enthesitis-related arthritis, psoriatic arthritis, and undifferentiated arthritis. Despite established classifications, the terms and definitions surrounding these conditions can often lead to confusion among healthcare professionals. This ambiguity underscores the need for a standardized approach to nosological classification. The clinical presentation of JSpAs can be multifaceted, encompassing both articular and extra-articular manifestations. Articular symptoms may include enthesitis and varying forms of arthritis, while extra-articular involvement can range from uveitis to gastrointestinal, cardiovascular, pulmonary, neurological, and renal complications. These diverse manifestations highlight the systemic nature of the disease and the importance of a holistic approach to diagnosis and treatment. While laboratory tests for SpAs are often non-specific, imaging modalities such as musculoskeletal ultrasound and magnetic resonance imaging play a crucial role in the early detection of inflammatory lesions. These imaging techniques can provide valuable insights into disease progression and aid in the formulation of appropriate treatment plans. Current treatment guidelines advocate for a “stepwise” approach to therapy, beginning with nonsteroidal anti-inflammatory drugs and progressing to glucocorticoids, disease-modifying antirheumatic drugs, and biological agents, particularly anti-tumor necrosis factor alpha agents. The primary objective of treatment is to achieve clinical remission or, at a minimum, to attain low disease activity. Regular monitoring of disease activity is imperative; however, the lack of validated assessment tools for the pediatric population remains a significant challenge. JSpAs pose unique challenges in terms of diagnosis and management due to their diverse manifestations and the complexities of their classification. Ongoing research and clinical efforts are essential to refine our understanding of these conditions, improve treatment outcomes, and enhance quality of life for affected children and their families. Effective management hinges on early detection, individualized treatment plans, and continuous monitoring, ensuring that patients receive the most appropriate care tailored to their specific needs.

## 1. Introduction

Spondyloarthropathies (SpAs) encompass a group of inflammatory, immune-mediated disorders characterized by distinct features that set them apart from other forms of inflammatory arthritis. These differences are evident in aspects such as genetic predisposition, etiopathogenesis, and the evolution of the disease. According to the classification criteria established by the International League of Associations for Rheumatology (ILAR) in 2004, juvenile idiopathic arthritis (JIA) can be categorized into seven distinct forms [1]. Notably, among these forms, arthritis associated with enthesitis, psoriatic arthritis, and undifferentiated arthritis falls under the umbrella of juvenile spondyloarthropathies (JSpAs) [2,3]. Additionally, patients diagnosed before the age of 16 who satisfy the modified New York criteria for ankylosing spondylitis (AS), the European Spondyloarthropathies Study Group (ESSG) or Amor criteria for undifferentiated arthritis, or the Assessment of the Spondyloarthritis International Society (ASAS)’s criteria for axial or peripheral spondyloarthritis are also classified as part of the JSpAs cohort [2,4]. This classification underscores the heterogeneous nature of SpAs and the importance of early diagnosis and tailored therapeutic approaches for affected pediatric populations.

The clinical manifestations of JSpA encompass both articular and extra-articular features, including enthesitis, peripheral or axial arthritis, uveitis, nail alterations, and gastrointestinal disturbances. Despite the presence of these symptoms, the clinical presentation remains largely nonspecific, often resulting in delayed diagnosis [4,5]. Laboratory investigations typically yield nonspecific results that may indicate chronic inflammation; while some patients may display a negative rheumatoid factor (RF) and a positive human leukocyte antigen (HLA)-B27, these findings are insufficient for an accurate diagnosis [2]. Conversely, imaging techniques such as radiography, musculoskeletal ultrasonography, and magnetic resonance imaging (MRI) play a crucial role in the diagnostic process and subsequent monitoring of patients exhibiting signs and symptoms consistent with JSpA [4]. The reliance on these advanced imaging resources underscores the need for a multifaceted approach to effectively identify and manage this complex condition.

In the realm of pediatric rheumatology, the treatment of JSpAs emphasizes the urgent need to mitigate articular inflammation at the earliest stages of diagnosis. This proactive approach is essential to prevent complications and minimize the risk of long-term sequelae. Achieving therapeutic success necessitates a multidisciplinary strategy that fosters effective collaboration among various stakeholders, including patients, parents, and a diverse team of health professionals. Key disciplines involved in this collaborative effort include pediatric rheumatology, physiotherapy, ophthalmology, and pediatric psychiatry, underscoring the complexity of the condition and the importance of integrated care [5]. The 2019 guidelines established by the American College of Rheumatology (ACR) advocate for a stepwise therapeutic protocol. This protocol initiates treatment with nonsteroidal anti-inflammatory drugs (NSAIDs) to address pain and inflammation. Should these initial measures prove insufficient, the guidelines recommend escalating therapy to include disease-modifying antirheumatic drugs (DMARDs), such as methotrexate (MTX) and sulfasalazine (SSZ), or biological agents targeting specific inflammatory pathways, including anti-tumor necrosis factor (TNF) therapies and interleukin (IL) inhibitors (anti-IL-17, anti-IL-12, and anti-IL-23) [6]. To effectively monitor the disease’s progression and therapeutic response, various tools have been developed to quantify disease activity. However, it is noteworthy that the disease activity index remains the sole tool specifically tailored for JSpA assessment. This index evaluates clinical parameters such as the presence of enthesitis, sacroiliitis, uveitis, and thoracolumbar spine involvement, providing a comprehensive framework for understanding disease dynamics and guiding treatment strategies [4].

This narrative review aims to provide a comprehensive overview of the current diagnostic methods, monitoring strategies, and therapeutic options available for JSpAs.

## 2. Definition and Classification

SpAs encompasses a diverse range of inflammatory diseases, characterized by their association with the HLA-B27 antigen. These pathologies predominantly affect the axial skeleton and sacroiliac joints, while also manifesting in various other clinical symptoms, including enthesitis, inflammatory back pain, dactylitis, nail dystrophies, psoriasis, acute anterior uveitis, and gastrointestinal symptoms associated with inflammatory bowel disease (IBD). The spectrum of SpAs is extensive, ranging from early undifferentiated forms to well-defined disorders such as AS. A hallmark of SpAs is the presence of inflammatory back pain and the progressive involvement of the axial skeleton, with a significant subset of patients (10–20%) experiencing initial symptoms before the age of 16. JSpAs represents the pediatric manifestation of SpAs and is now understood as chronic conditions that can persist into adulthood, particularly among children diagnosed with JIA. JSpAs primarily consists of seronegative diseases, frequently presenting with negative RF and often negative antinuclear antibodies (ANA). The genetic predisposition associated with these disorders is strongly linked to the HLA-B27 antigen. Typically, the onset occurs before the age of 16, and the condition is more prevalent in males. The clinical presentation may include arthritis during childhood and adolescence, enthesitis, and a risk of axial inflammatory involvement, often accompanied by radiographic evidence of sacroiliac joint damage. JSpAs can be categorized into differentiated forms, which include juvenile ankylosing spondylitis (JAS), psoriatic arthritis, reactive arthritis, and arthritis associated with IBD. Additionally, undifferentiated forms, such as seronegative enthesopathy and arthropathy syndrome (SEA) and enthesitis-related arthritis (ERA), are recognized within this classification. Collectively, these entities underscore the complexity and heterogeneity inherent in SpAs, necessitating a nuanced approach to diagnosis and management [5].

SpAs presents distinct challenges in pediatric populations, despite the clear classifications established for adults. JSpA is currently classified as a subtype of JIA, yet it possesses unique clinical characteristics that warrant special attention. Over the years, various classification systems have been proposed for both adult and pediatric SpA to foster a shared understanding, enabling effective management, early diagnosis, targeted therapies, and vigilant monitoring. According to the ASAS criteria, adult spondyloarthritis is categorized into peripheral and axial forms. The modified New York criteria further refine these classifications by incorporating both clinical and radiological features. In children, the ERA form is recognized as analogous to adult AS. To specifically define and classify JSpA, the ILAR criteria, as well as the guidelines established by the Pediatric Rheumatology International Trials Organization (PRINTO), are utilized. This comprehensive approach is vital for advancing our understanding and management of SpA across different age groups [3].

According to the ILAR, ERA is defined through a comprehensive set of inclusion and exclusion criteria, which are pivotal for the accurate diagnosis and effective management of the condition. The inclusion criteria entail that a patient must exhibit either arthritis and enthesitis, or at least one of these two manifestations—inflammation of the joints and entheses—accompanied by a minimum of two additional indicators. These indicators include the presence of sacroiliac joint tenderness or inflammatory lumbosacral pain, the presence of the HLA-B27 antigen, the onset of arthritis in male patients after the age of six, a family history of HLA-B27-associated disorders (such as AS, ERA, sacroiliitis related to IBD, reactive arthritis, or symptomatic acute anterior uveitis) in first-degree relatives, or the symptomatic occurrence of acute anterior uveitis. Conversely, the exclusion criteria delineate specific conditions that disqualify a diagnosis of ERA. These include the presence of psoriasis in the patient or in a first-degree relative, the detection of IgM RF on at least two separate occasions, spaced a minimum of three months apart, the diagnosis of systemic JIA in the patient, or a case where the arthritis meets the criteria for two distinct JIA categories [1,3,7]. Adhering to these criteria is essential for clinicians to ensure the accurate identification of ERA, guiding appropriate therapeutic interventions and improving patient outcomes.

ERA is characterized by its initial manifestation, predominantly in the form of peripheral involvement among children and adolescents. At the onset of the disease, patients typically present with inflammation, affecting the articulations and entheses of the lower extremities. Notably, axial involvement is relatively uncommon at this stage, often emerging only after the onset of peripheral arthritis. In contrast, axial SpA (axSpA) is primarily observed in adult populations, whereas peripheral SpA is more frequently diagnosed in younger patients, including children and adolescents. Imaging techniques, particularly MRI, play a critical role in the assessment of sacroiliac articulations in adults presenting with inflammatory back pain. This is especially relevant given that the early detection of inflammation via MRI can significantly impact treatment decisions. It is important to note that in cases of juvenile-onset SpA, inflammatory back pain occurs in less than 1% of patients during the initial years of disease. The presence of MRI-detected edema in the SI joints serves as a pivotal indicator for initiating therapy in adult-onset SpA. Research indicates that the timing of treatment commencement is crucial; earlier intervention is associated with a more favorable therapeutic response. This highlights the importance of timely and accurate diagnostic imaging in the management of both ERA and axial SpA, ultimately guiding clinicians in delivering effective care tailored to the unique presentations of these conditions in different age groups. Understanding these dynamics is essential for improving outcomes in patients affected by these forms of arthritis.

In 2019, Martini et al., in collaboration with PRINTO, introduced revised criteria for the classification of JIA. This innovative framework was developed based on a comprehensive synthesis of medical evidence, as well as clinical and paraclinical investigations routinely available in medical settings. The primary objective of these new criteria is to differentiate childhood-specific forms of JIA from those that parallel adult-onset conditions. Notably, it is recognized that all categories of adult-specific SpAs can manifest during childhood, particularly in the context of undifferentiated forms. Consequently, the term “enthesitis/spondylitis-related JIA” has been proposed to encompass these presentations, which include axial inflammatory damage and adhere to radiological criteria, specifically the identification of sacroiliitis through advanced imaging techniques. This advancement in classification not only enhances diagnostic accuracy, but also facilitates tailored therapeutic interventions for affected pediatric populations [8].

Numerous studies have examined the distinctions between the ILAR and the PRINTO classification criteria for JIA. In a comprehensive analysis involving a cohort of 1223 children diagnosed with JIA, it was observed that 65 patients with ERA were classified under the PRINTO criteria as enthesitis/spondylitis related. Notably, 59 of these patients, accounting for 90.8%, also satisfied the ILAR criteria for enthesitis-related JIA. However, among the children diagnosed with ILAR ERA who did not meet the PRINTO ERA criteria—comprising all male participants over the age of six—enthesitis or arthritis was present, but they only fulfilled one of the additional diagnostic requirements, which included HLA-B27 positivity, uveitis, spinal pain, or a positive family history. The primary differences between the two classification systems were identified in their respective lists of familial disease history, with the PRINTO criteria being more stringent, and the incorporation of new diagnostic elements such as inflammatory back pain and sacroiliitis observed through MRI in the PRINTO criteria [9].

In their comparative analysis of classification systems, Lee et al. observed a notable discrepancy between the ERA-ILAR criteria and the PRINTO classification. Specifically, the study revealed that only 60% of patients, amounting to 134 out of a total of 225 individuals who met the ERA-ILAR criteria, were subsequently classified as having ERA or spondylitis-related JIA according to the PRINTO classification [10]. This finding highlights the potential limitations and variances in the diagnostic criteria used in pediatric rheumatology, underscoring the importance of standardized classification systems for accurate diagnosis and targeted treatment of juvenile arthritis conditions.

In the research conducted by Akca et al., which involved a cohort of 108 patients diagnosed with ERA, the ASAS criteria for peripheral SpA exhibited superior sensitivity, at 85.1%. Conversely, the ILAR criteria for ERA and the PRINTO criteria demonstrated the highest specificity, reaching 100% in both diagnostic assessments and follow-up evaluations [7]. This finding is corroborated by the study of Catarino et al., which also concluded that the ASAS criteria are notably sensitive for peripheral SpA, whereas the ILAR and PRINTO criteria maintain the highest specificity for both peripheral and axial forms of ERA/spondylitis. The authors suggest that the ASAS axSpA criteria may facilitate the earlier detection of axial involvement. They advocate for the inclusion of imaging assessments for sacroiliitis in the definition of axial SpA, positing that this adjustment could significantly reduce the duration from disease onset to definitive diagnosis [11].

In 2024, a groundbreaking advancement in pediatric medicine was achieved with the introduction of the first-ever criteria specifically designed for the identification of axSpA in children. This initiative was spearheaded by an international consortium of experts who sought to address the unique clinical presentations and diagnostic challenges associated with axSpA in the pediatric population. The newly proposed criteria encompass seven distinct domains, categorized into three primary groups: one genetic domain, which considers a family history of SpA or the presence of the HLA-B27 antigen; four clinical domains that assess various aspects of axial pain, including frequency and chronicity, pain patterns, sacral and buttock pain, and morning stiffness; and two imaging domains focused on identifying active inflammation and structural lesions. The statistical analysis of these criteria revealed a remarkable specificity of 97.5%, indicating a high likelihood that individuals meeting these criteria truly have the condition. However, the sensitivity was calculated at 64.3%, suggesting that while the criteria are effective in confirming diagnoses, they may not capture all cases of pediatric axSpA. Notably, when compared to the existing criteria for adults, such as the ILAR-ERA, ASAS axial, and European Spondyloarthropathy Study Group criteria, the proposed pediatric criteria demonstrated superior specificity, further highlighting their potential utility in clinical practice [12]. This development represents a significant step forward in the accurate diagnosis and management of axSpA in children, paving the way for improved patient outcomes and tailored therapeutic approaches. In Table 1, we present a summary of the pediatric and adult classification criteria.

## 3. Epidemiology

The ERA form of JIA has been documented in various studies to affect between 8.6% and 18.9% of children diagnosed with the condition. Additionally, it is estimated that approximately 8.6% to 11% of adult cases of AS exhibit symptom onset during childhood. In the adult population, the prevalence of AS and related SpA conditions demonstrates a strong association with the HLA-B27 antigen across diverse demographics. The incidence of this antigen is estimated to be between 6% and 8% among Europeans, while it is observed to be less frequent in Japanese and African populations, with some reports suggesting an association between AS and other HLA molecules, such as HLA-B14:03 [13]. Research conducted in Europe indicates that the most prevalent category of JIA is oligoarthritis, with ERA accounting for only 10% to 16% of all JIA cases [14]. In contrast, data from Southeast Asia suggest a higher occurrence of both ERA and systemic arthritis. Notably, studies focused on the Chinese population have revealed that ERA may be the most common type of JIA, with reported prevalence rates ranging from 34.88% to 40% [15]. These findings highlight the variability in the prevalence of different JIA forms across geographic regions and underscore the importance of considering genetic and environmental factors in the epidemiology of these conditions.

ERA is a subtype of JIA that is notably more prevalent among the Chinese population. This increased incidence is likely attributable to the high frequency of the HLA-B27 antigen within this demographic. The average age for the diagnosis of ERA typically falls between 10 and 13 years, with a mean age of approximately 11.7 years. Interestingly, ERA is the only form of JIA that exhibits a higher prevalence in males compared to females. It is crucial to pay particular attention to the manifestations of ERA in females, as the clinical presentation in girls may differ significantly; symptoms often emerge later in life and tend to be less severe. Additionally, in female patients, axial involvement is more frequently observed than peripheral arthritis, highlighting the need for tailored diagnostic and therapeutic approaches in this subgroup of patients [1].

Recent epidemiological findings have been corroborated through an extensive analysis involving 902 pediatric patients diagnosed with ERA and psoriatic arthritis, all of whom were registered in the Childhood Arthritis and Rheumatology Research Alliance Registry from 2015 to 2018. The study revealed that the average age of diagnosis for ERA was notably higher, at 10.8 years, in comparison to juvenile psoriatic arthritis. Furthermore, the data indicated a higher prevalence of ERA among male patients, accounting for 56% of the cases. The clinical presentation was predominantly characterized by polyarticular involvement, observed in 57% of the participants. In terms of treatment, a significant 72% of the patients required intervention with at least one biological agent throughout the course of their illness. This was particularly pronounced in instances associated with sacroiliitis, which was reported in 40% of the patients, with females constituting 54% of this subgroup [16]. These findings underscore the critical need for tailored therapeutic approaches in managing pediatric arthritis and highlight the gender disparities observed in the disease’s manifestation.

In a study conducted by Ghantous et al. across four prominent pediatric rheumatology centers—three located in Israel and one in the United States—87 patients diagnosed with JSpA were evaluated to assess their demographic, clinical, and radiological characteristics. The findings indicated that the Israeli cohort exhibited a later onset of diagnosis compared to their American counterparts. Furthermore, the Israeli patients demonstrated a higher frequency of axial involvement, as evidenced by MRI findings, in contrast to peripheral arthritis or enthesitis. Notably, the prevalence of positive HLA-B27 cases was significantly lower among the Israeli patients [17]. These results underline the critical role that genetic and environmental factors may play in the etiopathogenesis of JSpA, an area that requires further elucidation to enhance our understanding of the disease’s complexities.

## 4. Etiopathogenesis

**Figure 1:**
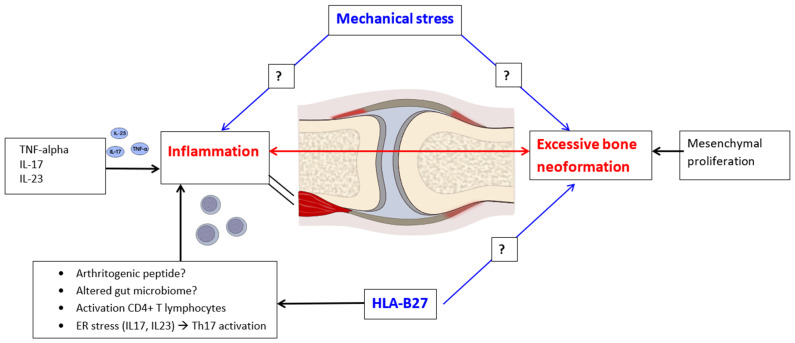
Schematic representation of the mechanisms of enthesitis in juvenile spondyloarthropathies. ER, endoplasmic reticulum; HLA, human leukocyte antigen; IL, interleukin; Th, T helper lymphocytes; and TNF, tumor necrosis factor.

JSpA is a chronic inflammatory condition that manifests through a local tissue response characterized by osteoarticular erosion and pathological bone neoformation. This condition bears similarities to the adult forms of SpAs, creating a distinct immune and anatomical microenvironment [18,19]. The primary structural alteration observed in JSpA involves bone remodeling, which is evidenced by excessive bone formation in the inflamed periarticular regions. Inflammation induces inhibitors of bone neoformation and causes the destruction of cartilage and bone. The decline in inflammation is followed by bone proliferation. This process is concomitant with accelerated systemic bone resorption and results in the development of syndesmophytes, enthesophytes, and ultimately, ankylosis [19]. On the other hand, there are new data suggesting the importance of repetitive microtrauma in promoting site-specific inflammation [19]. Enthesitis, a hallmark of JSpA, is predominantly driven by the pathways of TNF-alpha and IL-17/23. The presence of the HLA-B27 antigen appears to play a significant role in both inflammatory processes and bone neoformation (Figure 1). Evidence from studies focusing on HLA-B27-positive patients with JIA indicates that the most prevalent ILAR category is ERA. Furthermore, HLA-B27 positivity is associated with a more severe and chronic disease trajectory, necessitating a more aggressive medical intervention [20]. This highlights the importance of understanding the underlying immunological mechanisms and genetic predispositions in managing JSpA and tailoring therapeutic approaches.

Recent studies have presented controversial data indicating a potential link between gut dysbiosis and the interaction of HLA-B27 with the gut microbiome. This link suggests that alterations in the gut’s microbial composition may play a significant role in modulating the immune responses associated with HLA-B27. Specifically, gut microbes have been shown to activate the Toll-like receptor (TLR) pathway, which could subsequently lead to the activation of monocyte–macrophage lineage cells. This activation mechanism is critical, as it may contribute to the inflammatory processes often observed in conditions associated with HLA-B27 [21]. Studies revealed disturbances in the gut microbiome of ERA patients without any digestive symptom compared with healthy controls, with higher populations of bacteria from the genera *Bacteroides* (mainly *B. fragilis*, *B. plebeius*, and *B. eggerthii*), *Enterococcus*, and *Klebsiella* and a lower representation of the genus *Prevotella* (including *P. copri* and *P. stercorea*), which were not reversed by probiotic administration [22]. In the adult population with SpA, Berland et al. identified a negative correlation between the richness of the gut microbiota and disease activity [23]. The same study showed a significantly reduced gut microbiota diversity in HLA-B27-positive healthy controls compared to their HLA-B27-negative counterparts [23]. Such findings underscore the importance of further investigation into the gut microbiome’s influence on immune regulation and its implications for diseases linked to HLA-B27, including various forms of SpAs, especially regarding pediatric population.

In the context of SpA pathogenesis, recent research highlights three primary mechanisms through which the HLA-B27 antigen may exert its effects. Firstly, the activation of lymphocytes occurs via the presentation of an unidentified arthritogenic peptide, which initiates an immune response. Secondly, CD4-positive T lymphocyte activation is facilitated through dimerization on the surface of antigen-presenting cells, which further enhances the immune activation process. Lastly, the induction of endoplasmic reticulum (ER) stress leads to the secretion of pro-inflammatory cytokines, specifically IL-17 and IL-23, culminating in the activation of T helper 17 (Th17) lymphocytes. A prominent theory regarding the immunopathogenic role of HLA-B27 in SpA posits that the misfolding of this antigen within the ER causes cellular stress. However, it is important to note that various stimuli, including bacteria, fungi, prostaglandin E2, and IL-3, can also instigate the production of IL-23 via myeloid cells. This cytokine plays a dual role, acting as a driver of enthesitis and contributing to inflammation in structures analogous to the entheses, such as the ciliary body, thereby providing an explanation for the occurrence of associated uveitis [24]. Furthermore, early-stage SpA is characterized by a significant association of osteitis with low-grade IBD. This has been substantiated in pediatric patients with ERA, who exhibit elevated fecal calprotectin levels—an established surrogate marker for intestinal inflammation. Additionally, colonoscopy findings have revealed that SpA patients with subclinical inflammation are at a heightened risk of the progressive development of active arthritis, particularly involving the hip joints [18]. This insight holds considerable prognostic value, underscoring the interconnected nature of SpA and its potential complications.

The neutralization of IL-17A has exhibited substantial efficacy in managing conditions such as AS, psoriasis, and psoriatic arthritis; however, it has also been linked to the exacerbation and onset of IBD and colitis. This phenomenon may be attributed to the protective role of IL-17A within the gastrointestinal tract [25]. Concurrently, monoclonal antibodies targeting IL-23, such as risankizumab, as well as those directed against both IL-12 and IL-23, like ustekinumab, have demonstrated marked effectiveness in treating psoriasis, psoriatic arthritis, and Crohn’s disease. Yet, these treatments have shown limited efficacy in AS and axial SpA. While preclinical evidence strongly implicates the IL-23/IL-17 signaling axis in the pathophysiology of SpA, the ineffectiveness of anti-IL-23 therapies in axSpA suggests that IL-23 and IL-17 may have distinct roles at different stages of disease progression [4]. Furthermore, investigations into genetic predispositions have revealed single nucleotide polymorphisms (SNPs) in the ER aminopeptidase (*ERAP1*) and the IL-23 receptor (*IL23R*) genes in JIA. Notably, the *ERAP* allele C (rs27044) has been associated with a heightened risk of poor functional outcomes in univariate analyses [26]. Additionally, the IL-36 cytokine family, encompassing three isoforms (α, β, and γ), alongside its receptor antagonist IL-36Ra, has been implicated in the pathogenesis of psoriasis and IBD—conditions that can act as precursors to SpA. The evidence indicates that patients with JIA-ERA exhibit elevated levels of IL-36γ expression and serum concentrations, suggesting the potential role of IL-36 in the inflammatory processes associated with synovitis. Looking ahead, therapies based on IL-36Ra may hold promise for the treatment of ERA [21]. Lastly, a noteworthy study found that exclusive breastfeeding for more than six months was independently and significantly linked to a reduced risk of developing JSpA. This raises important questions for future research regarding the potential influence of early microbiome modulation on the subsequent development of JSpA [27]. Further investigation into these correlations may yield valuable insights into preventive strategies and therapeutic interventions for individuals at risk of developing SpA.

## 5. Clinical Picture

ERA presents a complex clinical profile that varies significantly among patients. The manifestations of this condition range from general systemic symptoms, such as fatigue and fever, to more specific musculoskeletal complaints, including pain and stiffness in the peripheral joints of the lower extremities. Notably, enthesitis affects 60–80% of patients, while axial spine pain can lead to reduced mobility. The principal signs and symptoms associated with ERA can be categorized into three distinct groups: enthesitis, arthritis (either peripheral or axial), and extra-articular manifestations, which encompass uveitis, gastrointestinal involvement, and issues within the central nervous system and cardio-pulmonary and renal systems. Enthesitis, characterized by inflammation at the sites where tendons and ligaments attach to the bone, stands as a cardinal clinical feature of ERA, observed in over half of the affected individuals. This particular manifestation differentiates ERA from other forms of JIA and may even signal the onset of the condition [1]. Common sites of enthesitis include the patellar ligament, the calcaneal insertion of the Achilles tendon, and the metatarsal or calcaneal insertion of the plantar fascia. Less frequently, enthesitis may occur in the pelvis, spinal regions, or upper limbs. Clinically, pelvic enthesitis can be present even in the absence of overt peripheral enthesitis, with MRI findings highlighting the relationship between pelvic enthesitis and sacroiliitis. The identification of pelvic enthesitis, as documented via radiological assessment, serves as a critical indicator of inflammatory activity within the body. The prevalence of enthesitis varies depending on the geographical region and the specific definitions employed for SpA, with estimates ranging from 37% in ERA cases to nearly 100% in SpA cohorts. Phenotypically, ERA can be classified into two primary subtypes. The peripheral phenotype, predominantly affecting patients aged ten years or younger, is often associated with a negative HLA-B27 status in 51% of cases, and is typified by tibiotarsal arthritis, along with enthesitis. Conversely, the axial phenotype primarily impacts male patients aged eleven years and older, often correlating with a positive HLA-B27 status. This phenotype is characterized by the involvement of the hip, sacroiliac joint, or lumbar spine, frequently accompanied by acute anterior uveitis or IBD, and typically exhibits a poorer response to DMARD, compared to a more favorable response to anti-TNF-alpha agents. In the early stages of ERA, joint damage commonly manifests as oligoarthritis or polyarthritis, particularly affecting the larger joints of the lower limbs. Consequently, these patients may initially be categorized under oligoarticular or polyarticular JIA. The distinguishing factors between ERA and other types of JIA are the presence of enthesitis and axial joint damage, such as sacroiliitis. Peripheral arthritis predominantly affects the lower extremities, including the knees, ankles, midfoot, and hips, often presenting as oligoarticular and asymmetrical in nature. This pattern is particularly prevalent among preadolescent boys, leading to diffuse pain that may spontaneously resolve, thereby delaying the definitive diagnosis [13]. In contrast, symmetrical polyarthritis, which is commonly observed in female patients, may suggest an alternate JIA classification. The axial skeleton is involved in 16–25% of JAS cases at the onset of the disease, with a further 45% developing axial involvement during the progression of the condition. Importantly, active sacroiliitis is frequently asymptomatic in approximately 65% of patients, and is often identified through imaging modalities such as MRI, with significant lumbosacral or buttock pain exacerbated by prolonged inactivity serving as a notable warning sign. A study conducted by Guo et al. involving a cohort of 105 children with ERA found that 54.29% exhibited an axial form of the disease, while 45.71% presented with a peripheral form, underscoring the varied clinical presentations of this complex condition [14]. Understanding the multifaceted nature of ERA is essential for accurate diagnosis and effective management, allowing clinicians to tailor treatment approaches that address the unique phenotypic characteristics and clinical manifestations associated with this form of arthritis.

Patients diagnosed with axial ERA are typically from an older demographic and exhibit significantly elevated levels of inflammatory markers compared to their counterparts. Notably, over one-third of individuals with asymptomatic axial ERA have been identified and confirmed through imaging methodologies. This study highlights a concerning trend: due to the absence of overt symptoms, patients with axial ERA experience an average diagnostic delay of approximately 10 months, a duration considerably longer than that observed in patients with the peripheral variant of the disease. It is important to note that, while axial involvement is predominantly observed in adults at disease onset, a significant number of children and adolescents with ERA initially present with peripheral manifestations, characterized by inflammation in the joints and/or lower limb entheses. Furthermore, inflammatory back pain—defined as pain persisting for more than three months—serves as a critical diagnostic criterion for adult axial disease; however, it is significantly less prevalent among children in the early stages of their illness [13]. The study conducted by Chan et al., which involved a cohort of 40 patients with ERA, revealed that 78% of patients displayed radiological evidence of sacroiliitis, with 81% of these cases exhibiting bilateral involvement. Alarmingly, 70% of the patients demonstrated radiological structural changes at the time when sacroiliitis was initially detected on imaging [15]. This underscores the necessity for timely diagnosis and intervention in managing axial ERA, particularly among younger populations, whose symptoms may present differently to those in adults (Table 2).

Uveitis associated with juvenile arthritis syndromes, specifically JAS and ERA, occurs in approximately 3–7% of affected patients. This form of uveitis is characterized as acute and symptomatic, with manifestations including ocular congestion, pain, and photophobia. These symptoms serve to differentiate it from the silent variant observed in oligoarticular JIA forms, particularly those with positive ANA. Typically, the uveitis in these cases is unilateral and recurrent, presenting a lower likelihood of long-term sequelae compared to other types of uveitis. Epidemiologically, a notable predominance of male patients and the presence of the HLA-B27 antigen are characteristic features associated with uveitis in ERA [14]. However, some studies have indicated a positive association between the risk of uveitis and ANA-positive female patients with ERA, suggesting that gender and immunological factors may influence the occurrence of this ocular complication. In addition to ocular symptoms, a range of extra-articular manifestations can occur in patients with ERA. These may include gastrointestinal symptoms such as chronic abdominal pain, diarrhea, hematochezia, and weight stagnation. Cardio-pulmonary abnormalities may manifest as aortic or mitral regurgitation, as well as myo-, peri-, or endocarditis. Respiratory complications can include a decreased forced vital capacity alongside increased residual volume and functional residual capacity. Central nervous system involvement may present as atlantoaxial subluxation, while renal complications can include papillary necrosis and IgA nephropathy. It is noteworthy that, while gastrointestinal symptoms often indicate an underlying IBD, the other aforementioned manifestations tend to be sporadic in pediatric and adolescent populations [28]. Fever is present in up to one-third of patients at the onset of the disease, and the clinical presentation can often mimic the systemic form of JIA, complicating diagnosis and management. In summary, understanding the complexities of uveitis in the context of JAS and ERA is crucial for healthcare professionals involved in the management of pediatric rheumatic diseases. The early recognition and interdisciplinary management of associated extra-articular manifestations are imperative for improving patient outcomes [29].

## 6. Laboratory Explorations

Currently, no definitive diagnostic tests exist for JSpA. While the HLA-B27 antigen is present in 60–70% of JSpA patients, and a similar prevalence is observed in JAS and ERA (80% and 60–80%, respectively), its presence in 6–8% of the general population, particularly among Caucasians, limits its diagnostic specificity [1]. Different HLA-B27 subtypes may show various associations with specific clinical features, but this is a subject of intense research and controversy. For example, HLA-B27:15 is associated with a younger age of disease onset compared to the HLA-B27:04 or HLA-B27:05 haplotypes [30,31].

Markers associated with other JIA subtypes, such as RF and ANA, are not useful in screening for JSpA; notably, RF is consistently negative in JSpA. While elevated acute-phase reactants and anemia may suggest chronic inflammation, routine laboratory investigations, including on complete blood count, biochemistry profile, urinalysis, erythrocyte sedimentation rate (ESR), and C-reactive protein (CRP), typically yield unremarkable results in most JSpA patients. Mou et al. revealed a tendency of higher inflammation markers (CRP and ESR) and disease activity scores in HLA-B27-positive JSA patients comparted to controls, statistically significant for the HLA-B27:04 haplotype [30]. However, significantly elevated acute-phase reactants and/or persistent anemia warrant the consideration of concomitant IBD, prompting the assessment of fecal calprotectin [1]. Lamot et al. reported a moderate correlation between fecal calprotectin levels and ERA disease activity, also demonstrating higher levels in ERA patients with MRI signs of sacroiliac inflammation than those without, emphasizing that a parallel inflammation in the musculoskeletal system and gut occurs in children with SpA [32].

Recent studies have explored novel inflammatory markers as potential indicators of axial spondyloarthritis (axial SpA) disease activity. The neutrophil-to-lymphocyte ratio and red blood cell distribution width have emerged as promising candidates in this context. A study encompassing 78 axSpA patients demonstrated high sensitivity (94.9%) and specificity (97.4%) for the lymphocyte-to-monocyte ratio (LMR), ESR, and CRP in assessing disease activity. Furthermore, a correlation has been observed between LMR and the radiographic stage of sacroiliitis, with LMR values exhibiting an inverse relationship to the severity of radiographic changes [33]. While elevated levels of myeloperoxidase 8/14 were noted in pediatric patients with ERA, the correlation with disease activity proved weak [29]. Biomarkers that investigate bone metabolic features, such as serum matrix metallopeptidase-3 (MMP-3), the soluble receptor activator of nuclear factor-κB ligand (sRANKL), and osteoprotegerin (OPG), were investigated in relation to disease progression. MMP-3 and sRANKL displayed significantly higher serum concentrations in JAS cases compared to in controls, with MMP-3 being positively related to disease activity, suggesting the enhanced osteoclast function and imbalance of the RANKL/OPG system in the inflammatory processes of JAS patients [34]. MicroRNAs are being intensely researched in different pathologies; some of them, such as miR-16, miR-125a-5p, miR-146a, miR-155, and miR-223, were described in association with JIA [35]. Particularly, the CC genotype of miR-146a (rs2910164 polymorphism) was associated with increased susceptibility to ERA, underlining the potential use of microRNAs as diagnostic or prognostic biomarkers in JSpA [36]. These findings suggest that while certain inflammatory markers show promise in assessing axSpA activity, further research is needed to validate their clinical utility and establish robust correlations with disease severity across diverse patient populations.

## 7. Imaging Investigations

Imaging techniques play a crucial role in the diagnosis and management of JSpA. Radiography, while useful for excluding pathologies such as malignancy and trauma, and for identifying osteoporosis or erosive cysts, offers limited sensitivity in detecting early inflammatory changes. Musculoskeletal ultrasound (MSK US) provides superior visualization of early inflammatory lesions within peripheral joints, tendons, bursae, and entheses, accurately depicting synovitis, tenosynovitis, bursitis, and enthesopathy. Furthermore, US synovitis scores correlate strongly with MRI findings and clinical disease activity markers in JIA [37]. MRI, particularly whole-body or pelvic MRI, excels at identifying bone edema in the sacroiliac (SI) joints and atlantooccipital regions. Specific MRI sequences allow for the assessment of bone edema (indicative of inflammation, even prior to erosion), articular cartilage integrity, spinal changes, synovial activity, and subchondral bone tissue, demonstrating superior diagnostic capabilities compared to clinical examination in early disease detection and monitoring, particularly within coxofemoral, sacroiliac, and vertebral joints. MRI of the SI joint allows for the detection of inflammatory and structural changes in the subchondral bone marrow not visible on conventional radiography (bone marrow edema, inflammation within erosion cavities, and fatty lesions). Table 3 summarizes the imaging techniques and their diagnostic changes that are suggestive of JSpA.

However, interpreting SI joint imaging in pediatric populations requires the careful consideration of developmental anatomy. Normal variations such as cortical irregularities (present in 57% of healthy children) and metaphyseal signal intensity can be misinterpreted as subchondral bone marrow edema [38], and the presence of thick cartilaginous growth plates may be mistaken for sacroiliitis. In children, due to the natural growth process, the appearance of the SI joint is more complex than in adults with age- and sex-related differences caused by the progressive ossification of the apophyses of the sacral wings (significantly earlier in girls than in boys). On the MRI, the incomplete ossified segmental and lateral apophyses of the sacral wings are visible. At the same time, the width of the joint space will depend on the amount of ossification. Due to those growth-related differences, the imagistic diagnosis of a sacroiliitis in children is more difficult than in adults [39]. Early-stage JSpA may manifest with sacroiliitis and lumbar spine lesions in the absence of clinical symptoms, warranting the use of contrast-enhanced imaging to detect inflammation, although this is not considered beneficial in adults compared to STIR sequences [14].

An essential element for active sacroiliitis is the bone marrow edema (BME). During the ossification process in growing children, the bone marrow signal varies. The signal changes on T2-weighted or short tau inversion recovery (STIR) MRI sequences can mimic BME, suggesting sacroiliitis and inducing the escalation of therapy. If the presence of BME is not clear and not located in the subchondral bone marrow, the high signal will not be interpreted as positive for diagnosis [39]. While bone marrow edema generally indicates active disease and informs therapeutic decisions, it is important to note that it can also result from mechanical overload, trauma, infection, or neoplasms. Difficulties can arise in interpreting the presence of small joint effusions without concomitant bone marrow edema [40].

The ASAS MRI consensus criteria define sacroiliitis as either a) two foci of bone marrow edema (BME)/osteitis within a single slice or b) one focus of BME/osteitis in two consecutive slices [41,42]. However, studies have reported a significant false-positive rate for BME, reaching up to 40% in individuals without axSpA, particularly when using less stringent criteria (e.g., BME in >2 SI joint quadrants or >2 consecutive slices). To mitigate these limitations, Maksymowych et al. proposed revised criteria for identifying active and structural lesions. For active lesions, they suggest the presence of BME in either >4 SI joint quadrants at any location or in the same location across >3 consecutive slices. Similarly, for definite structural lesions, their proposed criteria include the following: >3 SI joint quadrants with erosions, >5 quadrants with fatty lesions, erosions at the same location across >2 consecutive slices, fatty lesions at the same location across >3 consecutive slices, or the presence of a deep (≥1 cm) fatty lesion. These refined criteria aim to improve the accuracy of the MRI-based diagnosis of axSpA by reducing false-positive classifications of sacroiliitis [42].

Erosive changes in sacroiliac joints represent advanced axSpA and are associated with a poor prognosis, necessitating more aggressive therapeutic interventions. The diagnostic criteria for structural lesions of axSpA in children and adolescents differ significantly from those used in adults due to variations in lesion prevalence and the presence of mimicking conditions. The higher frequency of fat lesions and ankylosis observed in adults with longer disease duration contrasts with the relatively shorter disease course in children. The ASAS definition of erosion, characterized by full-thickness cortical loss and the loss of underlying bright T1 marrow signal, presents challenges in pediatric populations. The incomplete ossification of the cortex (lacking a distinct dark line on imaging) and low T1 signal in the immature marrow are physiological characteristics in children that can lead to the misinterpretation of erosions. Diagnosing structural damage as erosion is challenging, because (during ossification) the delineation of the joint space can be very difficult in children. Using the MRI adult definition will give many false-positive diagnoses of erosions in pediatric patients. Also, joint facet defects are normal variants that can be seen in healthy children and can be mis-interpreted as erosions [43]. Ongoing studies have the goal of developing reliable and clinically useful definitions for erosion in the pediatric population. To address this, Weiss et al. [12] proposed adjusted criteria for identifying axSpA lesions in a cohort of 243 JSpA patients. These criteria define inflammatory lesions as bone marrow edema in more than three sacroiliac (SIJ) quadrants across all SIJ MRI slices (sensitivity 98.6%, specificity 96.5%), structural lesions as erosions in more than three quadrants, sclerosis or fat lesions in more than two SIJ quadrants, or backfill or ankylosis in more than two joint halves across all SIJ MRI slices (sensitivity 98.6%, specificity 95.5%) [38]. Further validation of these revised criteria through an expert consensus is warranted.

Assessing treatment responses in JSpA necessitates a robust imaging evaluation. Several scoring systems have been developed to facilitate the objective quantification of sacroiliitis and monitor treatment efficacy. A reliable instrument is the Spondyloarthritis Research Consortium of Canada (SPARCC)’s scoring system, which objectively quantifies sacroiliitis by assessing bone marrow edema (BME) on MRI [4]. Specifically, the SPARCC system quantifies BME within the iliac and sacral bones via increased signal intensity on short tau inversion recovery (STIR) images, analyzing six consecutive oblique coronal slices through the SI joint. Further research has explored the JIA MRI-sacroiliac joint scoring system (JAMRIS-SIJ), a multi-component tool that semi-quantitatively assesses both inflammation and damage within the SIJ. This system prioritizes bone marrow edema and osteitis as key indicators of inflammation, weighting them 1.5 times more significantly than erosion cavity inflammation, 1.9 times more significantly than joint space fluid, and 5.3 times more significantly than enthesitis [40].

In the future, artificial intelligence (AI) can be a solution for the MRI evaluation of sacroiliac joints, which is a time-consuming examination and should be performed by a radiologist with extensive experience in the assessment of pediatric MRI. AI can play the role of triage for normal MRI, but needs an extensive database in order not to give many false-positive results, which must be subsequently validated by a radiologist. Currently, AI algorithms are used only for the MRI assessment of sacroiliac joints in adults. In a recent study, a deep learning tool for the detection of axSpA-associated abnormalities in MRI showed a sensitivity of 88% for detecting inflammatory changes indicative of axSpA [44]. At this moment, due to the age-related differences previously presented, it is difficult to create fully automated algorithms for BME detection in children [45]. Also, there are novel imaging techniques of the sacroiliac joint that are being explored, specifically MRI T2-mapping, diffusion-weighted imaging (DWI), and dual-energy computer tomography (CT) [46].

## 8. Therapeutic Options

The current guidelines for the management of JSpA advocate a stepwise therapeutic approach. Initial treatment options include NSAIDs, such as naproxen (15–20 mg/kg/day orally, up to 500 mg/day maximum) or indomethacin (1–3 mg/kg/day orally, up to 500 mg/day maximum). While NSAIDs offer effective pain relief, their efficacy in achieving disease control is limited, demonstrating a response rate of approximately 20–30% in patients with or without enthesitis. Glucocorticoids represent another therapeutic avenue; the intra-articular administration of triamcinolone acetate (0.5–1 mg/kg, up to 20–40 mg/dose maximum) may be beneficial, particularly in patients with limited peripheral joint involvement. Systemic glucocorticoids can be considered as a bridging therapy. Sacroiliac joint injections with glucocorticoids offer modest therapeutic benefit [47]. For patients with peripheral joint involvement, DMARDs such as SSZ (40–50 mg/kg/day orally, up to 2000–3000 mg maximum) or MTX (10–15 mg/m^2^/week orally or subcutaneously, up to 20 mg/week maximum) are indicated. In cases of persistent or insufficient response to initial therapies, biological agents, such as TNF-alpha inhibitors (e.g., etanercept 0.8 mg/kg/week subcutaneously, or adalimumab 24 mg/m^2^/week subcutaneously), may be considered, particularly in patients with peripheral and/or axial involvement. A common initial strategy involves combining an NSAID with a DMARD (MTX or SSZ), with escalation to biological therapy (TNF-alpha inhibitors) if the response is inadequate [6].

In a study of patients with ERA or other SpAs, the primary rationale cited by physicians for initiating TNF inhibitor therapy was the presence of active disease, as determined by physical examination (61%), exceeding the combined presence of both physical examination findings and abnormal imaging (14%) or abnormal imaging alone (24%) [47]. This underscores a significant reliance on clinical assessment in treatment decisions. Contrastingly, Ravelli et al. proposed an international consensus for JIA treatment [48], advocating for clinical remission—defined as the absence of inflammatory signs and symptoms—as the primary therapeutic goal, with minimal or reduced disease activity as a secondary objective. Their recommendations emphasize individualized patient management (with informed consent), regular disease activity assessment using validated tools, treatment frequency adjusted to disease activity and extra-articular manifestations, a target of at least 50% disease activity improvement within three months and clinical remission within six months, iterative treatment adjustments to achieve the primary goal, and ongoing monitoring post remission [48]. Despite these guidelines, a lack of universal consensus persists regarding the utilization of biomarkers and imaging techniques for the detection of subclinical inflammation, resulting in variability in the assessment criteria across clinical practice.

The 2019 ACR guidelines provide nuanced recommendations for the management of JIA, stratified according to the predominant clinical presentation. In JIA with sacroiliitis, a stepwise approach is advocated. NSAIDs constitute first-line therapy. Persistence of active disease warrants the addition of anti-TNF agents, with SSZ serving as an alternative if anti-TNF therapy is contraindicated. Glucocorticoids may be employed for a duration of less than three months as “bridging therapy” via oral administration, or as adjunctive intra-articular therapy. Similarly, in JIA with enthesitis, NSAIDs are the initial treatment modality. Continued disease activity may necessitate the introduction of anti-TNF agents, potentially in conjunction with short-term (less than three months) oral glucocorticoid “bridging therapy” [1].

Studies suggest that in cases of JIA accompanied by extra-articular manifestations, such as recurrent uveitis or IBD, infliximab and adalimumab demonstrate superior efficacy compared to etanercept [49,50]. Children diagnosed with ERA typically present with a limited number of affected joints. Their treatment protocols often mirror those used for patients exhibiting the oligoarticular phenotype. This approach underscores the importance of recognizing the similarities in clinical presentation and disease management strategies across these two subsets of JIA. By adopting analogous treatment methodologies, healthcare providers can effectively address the unique challenges posed by ERA while ensuring that the therapeutic interventions remain consistent with those employed in managing oligoarticular arthritis. As such, this alignment in treatment strategies not only facilitates a streamlined care process, but also enhances the overall outcomes for children grappling with these inflammatory joint conditions [47]. However, even with TNF inhibitor therapy, children with JSpA/ERA may experience poorer outcomes than those with other JIA subtypes. Furthermore, hip arthritis and sacroiliitis often prove resistant to TNF inhibitor therapy, highlighting a potential limitation in the ability of these agents to prevent disease progression in these specific locations [13]. Clinician-reported data indicate that treatment non-response is the primary driver for switching anti-TNF therapy (72% in one survey) [49]. Sacroiliitis emerges as a crucial determinant of treatment response, frequently underlying both primary non-response (45%) and treatment failure. In assessing anti-TNF failure specifically for sacroiliitis, sacroiliac joint imaging is considered the paramount factor influencing clinical decision-making by 65% of rheumatologists [49]. The overwhelming majority (87%) of rheumatologists opt for a second anti-TNF agent following initial treatment failure, while 62% transition to a different medication class after two unsuccessful anti-TNF therapies [49].

In cases where anti-TNF therapies are ineffective, the therapeutic decision is challenging. There is evidence that pediatric patients with inflammatory arthritis could benefit from treatments initially targeted toward adults with spondyloarthritis, due to similarities in pathogenesis and clinical manifestations. Using the adult data, studies of new therapies in JSpA can offer new opportunities. Recent studies have highlighted the potential benefits of employing monoclonal antibodies targeting interleukins (IL), specifically anti-IL-17 (such as secukinumab and ixekizumab) and anti-IL-12/23 (such as ustekinumab and briakinumab). Secukinumab, a fully human monoclonal antibody that inhibits IL-17A, has demonstrated a significantly prolonged time to flare-up compared to placebo in randomized controlled trials involving patients with ERA and juvenile psoriatic arthritis. In two controlled trials involving patients with ERA and juvenile psoriatic arthritis, by week 12, more than 30–40% of patients had achieved non-active disease and demonstrated a significantly prolonged time to flare-up [51,52]. Ruperto et al. enrolled 83 patients with ERA and obtained the therapeutic achievement of JIA ACR-30 and JIA ACR-70 in 90.4% (75/83) and 69.9% (58/83), respectively, with sustained improvement of signs and symptoms up to week 104 and a favorable safety profile [51]. In another investigation involving 52 patients aged between 2 and 18 years with active ERA and insufficient response to conventional therapies, the subcutaneous administration of secukinumab resulted in inactive disease status in over 30% of participants by the 12-week mark [52]. Furthermore, secukinumab has achieved a response rate exceeding 75% in the pediatric population with ERA, positioning it as a promising alternative to traditional anti-TNF treatments [53]. A retrospective study of ERA patients who had failed anti-TNF therapy reported significant improvement in JIA disease activity, measured using the Juvenile Arthritis Disease Activity Score (JADAS) after treatment with secukinumab [54]. There remain few publications evaluating the use of secukinumab in the JIA patient population, and even fewer evaluating its utilization in refractory ERA and juvenile psoriatic arthritis. In a study with seven ERA and two juvenile psoriatic arthritis cases, after one or two anti-TNF therapies failed (primary or secondary resistance to anti-TNF), secukinumab achieved inactive clinical disease [55]. Concluding on the previously presented data, secukinumab seems to offer an alternative to ERA patients who are not responding (primary or secondary resistance) to anti-TNF therapies. At the same time, compared to TNF inhibitor therapy, secukinumab can offer at least similar chances for biologically naïve patients of rapidly achieving the status of inactive clinical disease.

Concurrently, ixekizumab, another monoclonal antibody against IL-17A, is currently being evaluated in a multicenter, open-label clinical trial aimed at assessing its efficacy in treating ERA, including juvenile-onset AS [47]. While other biological agents such as rituximab (anti-CD20 monoclonal antibody), abatacept (an antagonist of the T lymphocyte costimulatory pathway), and tocilizumab (anti-IL-6 monoclonal antibody) have been explored in adult populations, their effectiveness appears to be comparatively diminished [56].

The landscape of therapeutic options for polyarticular JIA and adult axSpA has evolved significantly with the introduction of Janus kinase (JAK) inhibitors. Janus kinases (JAKs) are members of a family of protein tyrosine kinases (TYKs) that facilitate cell signaling (promote signal transduction). From the four isoforms (JAK1, JAK2, JAK3, and TYK2), JAK1 is the most important for the signaling of receptors activated by interleukin (IL) 6, IL-10, IL-11, IL-19, IL-20, IL-22, and interferon. In this therapeutic category, tofacitinib and baricitinib are classified as pan-JAK inhibitors, while upadacitinib is considered a highly selective JAK1 inhibitor. Unlike biologics, the JAK inhibitors can be orally administered. Regarding efficacy, from the perspectives of patient response, remission, and changes in steroid use, JAK inhibitors were found to be effective [57]. Recent approvals of JAK inhibitors have marked a pivotal advancement in the treatment of polyarticular JIA and adult AS. In an open-label phase of research, a notable 75% response rate was observed in 21 children with ERA treated with tofacitinib, underscoring its effectiveness as an oral therapeutic option with a well-established safety profile [29,58]. Furthermore, upadacitinib, another JAK inhibitor, demonstrated both efficacy and an acceptable safety profile in a cohort of 181 adult patients with AS [59]. In terms of safety, several studies reported that many adult patients experienced side effects, with infections being the most common. However, in pediatric patients, few serious adverse events were reported. Overall, three studies confirmed the safety of JAK inhibitors [60]. Recent advances have also introduced JAK inhibitors as a promising therapeutic option for patients with refractory JIA forms who do not respond to other biologic DMARDs. Another area of interest is the combination of JAK inhibitors with other biologics, such as IL-1 or IL-6 inhibitors, for patients with aggressive disease (for example refractory systemic JIA). Preliminary case reports suggest potential benefits, but more extensive trials are needed to evaluate their efficacy and safety [60]. At this moment, tofacitinib is approved by the FDA and EMA for children with active polyarticular JIA or juvenile psoriatic arthritis who are non-responsive to one or more DMARDs, but studies on its efficacy and safety in ERA are also ongoing. The efficacy of this drug in adults with ankylosing spondylitis is also promising for future approval in ERA.

**Figure 2:**
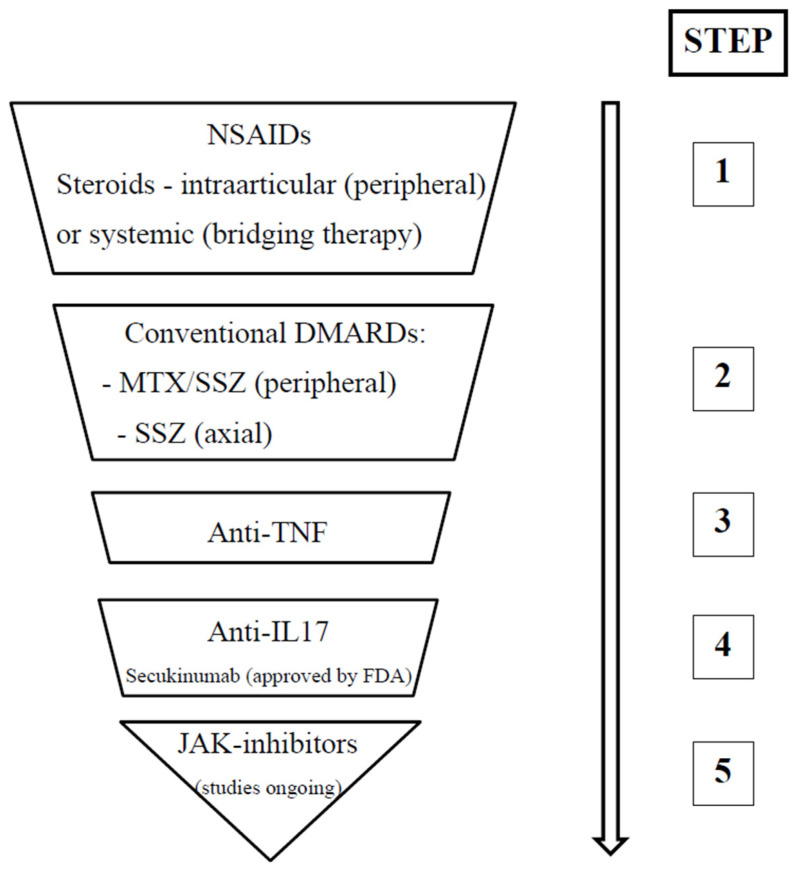
Step-up approach in ERA therapy. DMARDs, disease-modifying antirheumatic drugs; FDA, United States Food and Drug Administration; IL17, interleukin 17; JAK, Janus kinase; MTX, methotrexate; NSAIDs, non-steroidal anti-inflammatory drugs; SSZ, sulfasalazine; and TNF, tumor necrosis factor.

The promising outcomes associated with IL-17 inhibitors and JAK inhibitors suggest the emergence of novel therapeutic avenues for managing ERA, extending beyond traditional TNF inhibitors. Future research is warranted to determine whether these innovative agents will be preferred following TNF inhibitor failure, or if they can be utilized as first-line biologic therapies [47]. Importantly, a significant proportion of patients with ERA—approximately one-third to one-half—can develop “silent” axial disease, characterized by the absence of reported inflammatory back pain, while still exhibiting MRI evidence of acute disease or chronic destructive changes. Therefore, the necessity for future research to identify new clinical or biological markers correlating with axial involvement is imperative. An alternative strategy may involve more aggressive treatment approaches for patients with peripheral ERA, utilizing biologic therapy prior to the onset of axial disease [13]. Figure 2 summarizes the step-up approach regarding ERA therapy.

A meta-analysis encompassing 26 studies and 2354 JIA patients has illuminated the underlying causes of therapeutic failure and hypersensitivity to biological agents. Anti-drug antibodies have been identified with variable prevalence (0–82%) across nine biological agents, yielding an overall prevalence of 16.9% (95% CI 9.5–25.9). Notably, antibodies against infliximab, adalimumab, anakinra, and tocilizumab have been associated with therapeutic failure or hypersensitivity reactions. In cases where anti-drug antibody persistence is observed, potential solutions include immunosuppressive therapy, dosage adjustments, or a switch to alternative biological agents [61]. The advent of JAK inhibitors represents a significant milestone in the management of polyarticular JIA and adult AS, offering promising new options for patients. Ongoing research is critical to fully elucidate the roles of these agents in treatment paradigms and to develop strategies to mitigate the therapeutic failures related to anti-drug antibodies. The integration of innovative therapies into clinical practice holds the potential to enhance outcomes and quality of life for affected individuals.

## 9. Evolution, Monitoring, and Prognosis

From the perspective of the evolution and progression of ERA, it is observed that up to 40% of these cases may progress to AS, as delineated by the New York criteria. In instances where biological therapy is not utilized, clinical remission can be achieved in as many as 44% of patients; however, this remission is often accompanied by persistent enthesitis. Moreover, it is concerning that in 35% of cases, joint damage manifests, with the coxofemoral joint being the most frequently affected site. Notably, even in cohorts where the initial incidence of sacroiliitis was reported to be less than 30%, over half of the subjects developed axial disease within a five-year timeframe. Some studies indicate a potentially positive trend in the management of ERA, with up to 81% of patients attaining clinical remission within six months. Nonetheless, it is critical to note that 38% of these patients experienced flare-ups within the subsequent two years. On a global scale, this specific form of arthritis remains active in 30–50% of affected children, underscoring the pressing need for aggressive and continuous treatment strategies. Consequently, the cessation of therapy in these cases must be approached with greater caution compared to other forms of arthritis [13].

Monitoring disease activity in JIA and JSpAs is crucial for evaluating treatment responses and guiding therapeutic interventions. Among the various tools available, the JADAS has been validated specifically for the polyarticular and oligoarticular subtypes of JIA. However, its application in ERA remains limited, and it does not encompass critical aspects such as spinal damage or the presence of enthesitis and uveitis. The JADAS comprises six key parameters: the overall medical assessment of disease activity, the patient or parent’s general health assessment, functional capacity, the count of joints exhibiting active arthritis, the count of joints with restricted mobility, and levels of acute phase reactants (either ESR or CRP). In contrast, the Juvenile Spondyloarthritis Disease Activity (JSpADA) score has been specifically developed for patients with JSpAs, including those with JAS, ERA, psoriatic arthritis, and undifferentiated JIA. This tool stands out as the first validated instrument for assessing disease activity in the pediatric population with spondyloarthritis, featuring eight quantifiable parameters, each rated on a 0/1 scale. These parameters include the number of active arthritis joints, the presence of active enthesitis, duration of morning stiffness exceeding 15 min, inflammatory markers (ESR or CRP), clinical sacroiliitis presence, uveitis, self-reported pain by the patient, and spinal mobility as assessed by the Schober test [62]. Research indicates that a persistently elevated JSpADA score at follow-up is a significant predictive indicator of active disease six months later [63]. Thus, these tools play a pivotal role in the ongoing assessment and management strategies for pediatric patients suffering from these debilitating conditions, underscoring the necessity for healthcare providers to incorporate them into routine clinical practice.

In recent studies, various assessment tools designed for adult populations, such as the Bath Ankylosing Spondylitis Disease Activity Index (BASDAI) and the Ankylosing Spondylitis Disease Activity Score (ASDAS) with ESR, have demonstrated validation for use in patients with ERA. Moreover, the Bath Ankylosing Spondylitis Functional Index (BASFI) has shown promising efficacy in evaluating functional outcomes among individuals diagnosed with ERA. These instruments not only facilitate the monitoring of disease activity, but also enable healthcare professionals to assess the functional impact of the condition on patients’ daily lives. The application of these validated indices is crucial for enhancing the clinical management of ERA and improving patient outcomes [29]. The prognosis for patients with ERA is notably poorer when compared to other forms of JIA. This is evidenced by lower rates of remission, increased pain levels, and diminished quality of life. Once axial lesions manifest, the efficacy of available therapeutic options, including biological agents, may be compromised, complicating disease management. In a cohort study involving 73 HLA-B27-positive patients with ERA, several predictors of poor outcomes were identified in univariate analysis. These included the presence of inflammatory back pain, progressive imaging-confirmed sacroiliitis, and elevated CRP levels [41]. The presence of sacroiliitis and HLA-B27 has been shown to be associated with a chronic disease course and adverse outcomes. Interestingly, the early initiation of biological therapies appears to be associated with improved long-term prognoses, underscoring the importance of timely intervention [29]. The assessment of disease activity and functional impact through validated indices, combined with an understanding of prognostic factors, is crucial for optimizing management strategies for patients with ERA. Continued research into the identification of these predictors and the efficacy of early treatment interventions will be vital in improving outcomes for individuals suffering from this challenging condition.

Recent studies highlight the critical role that delayed diagnosis plays in the prognosis of ERA. In a cohort comprising 181 ERA patients, the average delay in diagnosis ranged from 1 to 5 years. This substantial delay was linked to significant functional impairment, with approximately 50% of patients demonstrating poor functional outcomes after a follow-up duration of 7 years. Notably, each additional year of diagnostic delay was associated with a 20% increased risk of experiencing adverse functional outcomes [26]. Expanding upon this theme, research conducted by Naveen et al. revealed that radiological hip involvement was associated with poor long-term outcomes. Alarmingly, nearly 90% of patients in this cohort exhibited active disease during adulthood, particularly in the context of the limited availability of biologic therapy. In a separate German cohort consisting of 118 treated ERA patients, it was found that 57% continued to experience persistent active disease into adulthood, while only 23% achieved medication-free remission [41]. Distinct from adults diagnosed with AS, children with JAS often experience the more severe involvement of the coxofemoral joints, resulting in a higher frequency of hip replacement surgeries. A comprehensive review of two studies that examined 307 patients and a total of 479 hips over a 23-year period (2000–2023) indicated that the majority of surgical interventions took place during the second and third decades of life. Additionally, patients suffering from tumors, skeletal dysplasia, and JIA demonstrated elevated rates of requiring custom-made implants [64]. Furthermore, patients with ERA—potentially due to the ramifications of immunosuppressive therapy—are at an increased risk of both early medical and surgical complications following total hip arthroplasty (THA). A retrospective cohort study involving 763 JIA patients who underwent THA illustrated that these individuals faced heightened risks of complications, including pneumonia, urinary tract infections, periprosthetic joint infections, periprosthetic fractures, aseptic loosening, dislocation, and the need for debridement or implant retention procedures [65]. This body of evidence underscores the importance of timely diagnosis and effective management strategies for ERA and JAS, as delays in diagnosis not only exacerbate the disease, but also significantly impact the long-term health outcomes and quality of life for affected individuals. As the field continues to evolve, addressing these challenges will be pivotal in improving patient care and outcomes.

## 10. Conclusions

SpAs encompasses a range of inflammatory pathogenic conditions that are closely associated with the HLA-B27 antigen. These conditions primarily target the axial skeleton and SI joints, leading to significant clinical implications. In particular, the presentation of arthritis and enthesitis in the lower extremities, or inflammatory back pain in adolescents—especially males—should heighten clinical suspicion for JSpAs. Typically, JSpA manifests initially as asymmetric oligoarticular arthritis, but its progression may include the early involvement of the axial skeleton, often occurring in the absence of overt symptoms. In addition to the arthritic features observed in the lower limbs and sacroiliac regions, patients may present with several common clinical manifestations, including enthesitis, inflammatory back pain, psoriatic skin lesions, dactylitis, and uveitis. While laboratory findings related to JSpA are generally nonspecific, imaging studies play a crucial role in both the diagnosis and ongoing monitoring of the disease’s progression. The importance of early intervention cannot be overstated, as the timely initiation of treatment is vital for optimizing long-term outcomes. A stepwise therapeutic approach is recommended, beginning with NSAIDs, followed by corticosteroids, DMARDs, or biologic therapies, depending on the severity of the disease and response to initial treatments. Through this comprehensive management strategy, clinicians can significantly improve the quality of life of patients affected by JSpA, mitigating the associated long-term complications.

The presented narrative review highlighted novel diagnostic and therapeutic advances in the field of JSpAs, but there are still certain areas that require further research, including the role played by genetic and environmental factors in the etiopathogenesis of JSpA, specific biomarkers for diagnostic and disease evolution monitoring, unifying criteria for more accurate imaging interpretation, or novel therapeutic strategies. This research is needed in order to provide more personalized disease management for patients suffering from this challenging condition.

## Figures and Tables

**Table 1:** Comparison between the classification criteria used for diagnostic establishment in children with spondyloarthropathies [1,8,12].

ILAR Criteria for ERA	PRINTO Criteria for E/SpRA	ASAS Criteria for pSpA	ASAS Criteria for axSpA	Weiss et al. [12] Score for axJSpA
Inclusion criteria: Arthritis + enthesitis OR Arthritis/enthesitis + ≥2 of the following: - sacroiliac joint tenderness/inflammatory lumbosacral pain - positive HLA-B27 - onset of arthritis in males >6 years - family history of HLA-B27-associated disorders (AS, ERA, sacroiliitis related to IBD, reactive arthritis, or anterior uveitis) in first-degree relatives - symptomatic acute anterior uveitis Exclusion criteria: History of psoriasis in the patient or a first-degree relative Detection of IgM RF on at least two separate occasions, minimum 3 months apart Criteria for systemic JIA Criteria for two distinct JIA categories	Peripheral arthritis + enthesitis OR Arthritis/enthesitis + ≥3 months of inflammatory back pain + sacroiliitis on imaging * OR Arthritis/enthesitis + ≥2 of the following: - sacroiliac joint tenderness - inflammatory back pain - positive HLA-B27 - symptomatic acute anterior uveitis - family history of SpA in first-degree relatives * defined as follows: - on radiograph: at least grade 2 bilateral sacroiliitis - on MRI: bone marrow edema on a T2-weighted sequence/bone marrow contrast enhancement on a T1-weighted sequence; inflammation clearly present and located in the subchondral bone; MRI appearance highly suggestive of SpA	Arthritis/enthesitis/dactylitis AND ≥1 of the following: - uveitis - psoriasis - IBD - preceding infection - positive HLA-B27 - sacroiliitis on imaging OR ≥2 of the following: - arthritis - dactylitis - enthesitis - history of inflammatory back pain - family history of SpA	≥3 months back pain + <45 years AND Sacroiliitis on imaging + ≥1 of the SpA features * OR Positive HLA-B27 + ≥2 of the other SpA features * * SpA features: - arthritis - uveitis - enthesitis - dactylitis - psoriasis - IBD - good response to NSAIDs - family history of SpA - positive HLA-B27 - elevated CRP	Imaging–evidence of active lesions typical of sacroiliitis on MRI (23) Imaging–structural lesions - evidence of sacroiliitis on radiograph (13) - evidence of structural lesions typical of sacroiliitis on MRI (23) Pain–chronicity - ≥4 days per week for ≥6 but <12 weeks (6) - ≥4 days per week for ≥12 weeks (9) Pain–pattern - awakens patient second half of the night/insidious onset (6) - moderate to total relief with NSAIDs (10) - improves with activity (13) Pain–location - patient reported lumbar spine pain (6) - sacroiliac pain with deep palpation or maneuvers/patient reported groin or hip pain (11) - patient reported sacral or buttock pain (12) Morning stiffness ≥15 min (9) Genetics - SpA or HLA-B27- associated acute symptomatic anterior uveitis in first-degree relative (8) - positive HLA-B27 (11) The score ≥55 is diagnostic

AS, ankylosing spondylitis; ASAS, Assessment of Spondyloarthritis International Society; axJSpA, axial juvenile spondyloarthritis; axSpA, axial spondyloarthritis; CRP, C-reactive protein; ERA, enthesitis-related arthritis; E/SpRA, enthesitis/spondylitis-related arthritis; IBD, inflammatory bowel disease; ILAR, International League of Associations for Rheumatology; JIA, juvenile idiopathic arthritis; MRI, magnetic resonance imaging; NSAIDs, nonsteroidal anti-inflammatory drugs; PRINTO, Pediatric Rheumatology International Trials Organization; pSpA, peripheral spondyloarthritis; RF, rheumatoid factor; and SpA, spondyloarthropathy.

**Table 2:** Differences in clinical presentation and evolution of symptoms between juvenile-onset and adult-onset spondyloarthropathies.

Disease Duration	Juvenile-Onset SpA	Adult-Onset SpA
<5 years	Clinical presentation: - Peripheral arthritis - Enthesitis - Inflammatory back pain ** Imaging: - Active sacroiliitis on MRI *	Clinical presentation: - Inflammatory back pain - Peripheral arthritis ** - Enthesitis ** Imaging: - Active sacroiliitis on MRI
5–10 years	Clinical presentation: - Peripheral arthritis - Enthesitis - Inflammatory back pain Imaging: - Sacroiliitis on radiography	Clinical presentation: - Inflammatory back pain - Peripheral arthritis * - Enthesitis * Imaging: - Sacroiliitis on radiography
>10 years	Clinical presentation: - Peripheral arthritis - Enthesitis - Inflammatory back pain Imaging: - Sacroiliitis on radiography	Clinical presentation: - Inflammatory back pain - Peripheral arthritis * - Enthesitis * Imaging: - Syndesmophytes

* Less frequent compared to the other categories; ** rare; MRI, magnetic resonance imaging; and SpA, spondyloarthropathy.

**Table 3:** Summary of imaging techniques and their diagnostic changes suggestive for juvenile spondyloarthropaty.

Site	Imaging Technique	Diagnostic Changes
Sacroiliac joint	Pelvic radiography	Diffuse osteoporosis, erosions, changes in joint width, sclerosis, and ankylosis;
	MRI	Periarticular bone marrow edema, sclerosis, fatty metaplasia of the bone, erosions, and fusion;
Spine	Radiography	Erosions at the corners of vertebral bodies with reactive sclerosis, vertebral body squaring, syndesmophytes, ossification of spinal ligaments, joints, and disks, and “bamboo spine” appearance;
	MRI	Early inflammatory changes in the vertebral body corner and edema within the subchondral bone;
Enthesis	Radiography	Bony abnormalities and abnormalities at the enthesis insertion sites;
	Ultrasonography	Hypoechogenicity, increased thickness, calcifications, enthesophytes, and increased Doppler activity;
Peripheral joints	Ultrasonography	Hypertrophy of the synovial membrane, hypoechoic synovial membrane, increased Doppler activity, and joint effusion;
	MRI	Synovial hypertrophy, synovial enhancement, erosions, subchondral cysts, and subchondral bone marrow edema.

MRI, magnetic resonance imaging.

## References

[1] Tse S.M., Colbert R.A., Petty R.E., Laxer R.M., Lindsley C.B., Wedderburn L.R., Mellins E.D., Fuhlbrigge R.C. (2021). Enthesitis-Related Arthritis. Textbook of Pediatric Rheumatology.

[2] Srinivasalu H., Treemarcki E.B., Redmond C. (2021). Advances in Juvenile Spondyloarthritis. Curr. Rheumatol. Rep..

[3] Fisher C., Ciurtin C., Leandro M., Sen D., Wedderburn L.R. (2021). Similarities and Differences Between Juvenile and Adult Spondyloarthropathies. Front. Med..

[4] Srinivasalu H., Sikora K.A., Colbert R.A. (2021). Recent Updates in Juvenile Spondyloarthritis. Rheum. Dis. Clin. N. Am..

[5] Yıldız M., Haşlak F., Adroviç A., Şahin S., Barut K., Kasapçopur Ö. (2022). Juvenile Spondyloartropathies. Eur. J. Rheumatol..

[6] Ringold S., Angeles-Han S.T., Beukelman T., Lovell D., Cuello C.A., Becker M.L., Colbert R.A., Feldman B.M., Ferguson P.J., Gewanter H. (2019). 2019 American College of Rheumatology/Arthritis Foundation Guideline for the Treatment of Juvenile Idiopathic Arthritis: Therapeutic Approaches for Non-Systemic Polyarthritis, Sacroiliitis, and Enthesitis. Arthritis Rheumatol..

[7] Kaya Akca U., Batu E.D., Sener S., Balik Z., Kasap Cuceoglu M., Atalay E., Basaran O., Bilginer Y., Ozen S. (2022). The Performances of the ILAR, ASAS, and PRINTO Classification Criteria in ERA Patients: A Comparison Study. Clin. Rheumatol..

[8] Martini A., Ravelli A., Avcin T., Beresford M.W., Burgos-Vargas R., Cuttica R., Ilowite N.T., Khubchandani R., Laxer R.M., Lovell D.J. (2019). Toward New Classification Criteria for Juvenile Idiopathic Arthritis: First Steps, Pediatric Rheumatology International Trials Organization International Consensus. J. Rheumatol..

[9] Shoop-Worrall S.J.W., Macintyre V.G., Ciurtin C., Cleary G., McErlane F., Wedderburn L.R., Chieng A., Ciurtin C., Baildam E., McErlane F. (2024). Overlap of International League of Associations for Rheumatology and Preliminary Pediatric Rheumatology International Trials Organization Classification Criteria for Nonsystemic Juvenile Idiopathic Arthritis in an Established UK Multicentre Inception Coho. Arthritis Care Res..

[10] Lee J.J.Y., Eng S.W.M., Guzman J., Duffy C.M., Tucker L.B., Oen K., Yeung R.S.M., Feldman B.M. (2022). A Comparison of International League of Associations for Rheumatology and Pediatric Rheumatology International Trials Organization Classification Systems for Juvenile Idiopathic Arthritis Among Children in a Canadian Arthritis Cohort. Arthritis Rheumatol..

[11] Catarino S., Nunes J., Ganhão S., Aguiar F., Rodrigues M., Brito I. (2024). Application of the New PRINTO Classification Criteria for Juvenile Idiopathic Arthritis in a Sample of Portuguese Patients. ARP Rheumatol..

[12] Weiss P.F., Brandon T.G., Aggarwal A., Burgos-Vargas R., Colbert R.A., Horneff G., Laxer R.M., Minden K., Ravelli A., Ruperto N. (2024). Classification Criteria for Axial Disease in Youth With Juvenile Spondyloarthritis. Arthritis Rheumatol..

[13] Smith J.A., Burgos-Vargas R. (2021). Outcomes in Juvenile-Onset Spondyloarthritis. Front. Med..

[14] Guo Y., Fang Y., Zhang T., Pan Y., Wang P., Fan Z., Yu H. (2023). Axial Involvement in Enthesitis-Related Arthritis: Results from a Single-Center Cohort. Pediatr. Rheumatol..

[15] Chan O.M., Lai B.M.H., Leung A.S.Y., Leung T.F., Ho A.C.H. (2023). High Prevalence of Sacroiliitis and Early Structural Changes in the Sacroiliac Joint in Children with Enthesitis-Related Arthritis: Findings from a Tertiary Centre in Hong Kong. Pediatr. Rheumatol..

[16] Rumsey D.G., Lougee A., Matsouaka R., Collier D.H., Schanberg L.E., Schenfeld J., Shiff N.J., Stoll M.L., Stryker S., Weiss P.F. (2021). Juvenile Spondyloarthritis in the Childhood Arthritis and Rheumatology Research Alliance Registry: High Biologic Use, Low Prevalence of HLA–B27, and Equal Sex Representation in Sacroiliitis. Arthritis Care Res..

[17] Ghantous N., Heshin-Bekenstein M., Dequattro K., Lakovsky Y., Hendel A.M., Rappoport N., Aviel Y.B., Tirosh I., Harel L., Weiss P.F. (2021). Do Geography and Ethnicity Play a Role in Juvenile Spondyloarthritis? A Multi-Center Binational Retrospective Study. Pediatr. Rheumatol..

[18] Harjacek M. (2021). Immunopathophysiology of Juvenile Spondyloarthritis (JSpA): The “Out of the Box” View on Epigenetics, Neuroendocrine Pathways and Role of the Macrophage Migration Inhibitory Factor (MIF). Front. Med..

[19] Tay S.H., Yeo J.G., Leong J.Y., Albani S., Arkachaisri T. (2021). Juvenile Spondyloarthritis: What More Do We Know About HLA-B27, Enthesitis, and New Bone Formation?. Front. Med..

[20] Peltoniemi S.O.O., Glerup M., Lahdenne P., Eklund K.K., Aalto K. (2023). Disease Characteristics of HLA-B27 Positive and Negative Finnish Patients with Juvenile Idiopathic Arthritis—Results of the 18-Year Cohort Follow-up Study. Pediatr. Rheumatol..

[21] Majumder S., Guleria S., Aggarwal A. (2022). IL-36γin Enthesitis-Related Juvenile Idiopathic Arthritis and Its Association with Disease Activity. Clin. Exp. Immunol..

[22] Aggarwal A., Sarangi A.N., Gaur P., Shukla A., Aggarwal R. (2017). Gut Microbiome in Children with Enthesitis-Related Arthritis in a Developing Country and the Effect of Probiotic Administration. Clin. Exp. Immunol..

[23] Berland M., Meslier V., Berreira Ibraim S., Le Chatelier E., Pons N., Maziers N., Thirion F., Gauthier F., Plaza Oñate F., Furet J.P. (2023). Both Disease Activity and HLA–B27 Status Are Associated With Gut Microbiome Dysbiosis in Spondyloarthritis Patients. Arthritis Rheumatol..

[24] Bridgewood C., Sharif K., Sherlock J., Watad A., McGonagle D. (2020). Interleukin-23 Pathway at the Enthesis: The Emerging Story of Enthesitis in Spondyloarthropathy. Immunol. Rev..

[25] Deng Z., Wang S., Wu C., Wang C. (2023). IL-17 Inhibitor-Associated Inflammatory Bowel Disease: A Study Based on Literature and Database Analysis. Front. Pharmacol..

[26] Ravichandran N., Guleria S., Mohindra N., Aggarwal A. (2023). Predictors of Long-Term Functional Outcomes of Juvenile Idiopathic Arthritis-Enthesitis-Related Arthritis: A Single Centre Experience. Rheumatology.

[27] Baggett K.H., Brandon T.G., Xiao R., Weiss P.F. (2024). Association of Infant Breastfeeding and Juvenile Spondyloarthritis: A Case-Control Study. J. Rheumatol..

[28] Pagnini I., Scavone M., Maccora I., Mastrolia M.V., Marrani E., Bertini F., Lamot L., Simonini G. (2021). The Development of Extra-Articular Manifestations in Children With Enthesitis-Related Arthritis: Natural Course or Different Disease Entity?. Front. Med..

[29] Naveen R., Guleria S., Aggarwal A. (2023). Recent Updates in Enthesitis-Related Arthritis. Rheumatol. Int..

[30] Mou Y., Zhang P., Li Q., Lin Z., Liao Z., Wei Q., Gu J. (2015). Clinical Features in Juvenile-Onset Ankylosing Spondylitis Patients Carrying Different B27 Subtypes. Biomed. Res. Int..

[31] Khan M.A. (2017). An Update on the Genetic Polymorphism of HLA-B*27 With 213 Alleles Encompassing 160 Subtypes (and Still Counting). Curr. Rheumatol. Rep..

[32] Lamot L., Miler M., Vukojević R., Vidović M., Lamot M., Trutin I., Gabaj N.N., Harjaček M. (2021). The Increased Levels of Fecal Calprotectin in Children With Active Enthesitis Related Arthritis and MRI Signs of Sacroiliitis: The Results of a Single Center Cross-Sectional Exploratory Study in Juvenile Idiopathic Arthritis Patients. Front. Med..

[33] Wang J., Su J., Yuan Y., Jin X., Shen B., Lu G. (2021). The Role of Lymphocyte-Monocyte Ratio on Axial Spondyloarthritis Diagnosis and Sacroiliitis Staging. BMC Musculoskelet. Disord..

[34] Mou Y.K., Zhang P.P., Li Q.X., Lin Z.M., Liao Z.T., Wei Q.J., Gu J.R. (2015). Changes of Serum Levels of MMP-3, SRANKL, and OPG in Juvenile-Onset Ankylosing Spondylitis Patients Carrying Different HLA-B27 Subtypes. Clin. Rheumatol..

[35] Orczyk K., Smolewska E. (2021). The Potential Importance of MicroRNAs as Novel Indicators How to Manage Patients with Juvenile Idiopathic Arthritis More Effectively. J. Immunol. Res..

[36] Singh S., Rai G., Aggarwal A. (2014). Association of MicroRNA-146a and Its Target Gene IRAK1 Polymorphism with Enthesitis Related Arthritis Category of Juvenile Idiopathic Arthritis. Rheumatol. Int..

[37] Sande N.K., Kirkhus E., Lilleby V., Tomterstad A.H., Aga A.B., Flatø B., Bøyesen P. (2024). Validity of an Ultrasonographic Joint-Specific Scoring System in Juvenile Idiopathic Arthritis: A Cross-Sectional Study Comparing Ultrasound Findings of Synovitis with Whole-Body Magnetic Resonance Imaging and Clinical Assessment. RMD Open.

[38] Weiss P.F., Brandon T.G., Lambert R.G., Biko D.M., Chauvin N.A., Francavilla M.L., Jaremko J.L., Herregods N., Kasapcopur O., Yildiz M. (2023). Data-Driven Magnetic Resonance Imaging Definitions for Active and Structural Sacroiliac Joint Lesions in Juvenile Spondyloarthritis Typical of Axial Disease: A Cross-Sectional International Study. Arthritis Care Res..

[39] Herregods N., Maksymowych W.P., Jans L.B.O., Otobo T.M., Sudoł-Szopińska I., Meyers A.B., Van Rossum M.A.J., Kirkhus E., Panwar J., Appenzeller S. (2021). Atlas of MRI Findings of Sacroiliitis in Pediatric Sacroiliac Joints to Accompany the Updated Preliminary OMERACT Pediatric JAMRIS (Juvenile Idiopathic Arthritis MRI Score) Scoring System: Part I: Active Lesions. Semin. Arthritis Rheum..

[40] Otobo T.M., Tolend M., Meyers A.B., Sudol-Szopinska I., Joshi S., Stimec J., Herregods N., Jaremko J.L., Tse S.M.L., Haroon N. (2023). Determination of Relative Weightings for Sacroiliac Joint Pathologies in the OMERACT Juvenile Arthritis Magnetic Resonance Imaging Sacroiliac Joint Score. J. Clin. Med..

[41] Naveen R., Mohindra N., Jain N., Majumder S., Aggarwal A. (2021). Hip Involvement in Children with Enthesitis Related Arthritis (ERA) Is Associated with Poor Outcomes in Adulthood. Clin. Rheumatol..

[42] Maksymowych W.P., Lambert R.G., Baraliakos X., Weber U., MacHado P.M., Pedersen S.J., De Hooge M., Sieper J., Wichuk S., Poddubnyy D. (2021). Data-Driven Definitions for Active and Structural MRI Lesions in the Sacroiliac Joint in Spondyloarthritis and Their Predictive Utility. Rheumatology.

[43] Herregods N., Maksymowych W.P., Jans L.B.O., Otobo T.M., Sudoł-Szopińska I., Meyers A.B., Van Rossum M.A.J., Kirkhus E., Panwar J., Appenzeller S. (2021). Atlas of MRI Findings of Sacroiliitis in Pediatric Sacroiliac Joints to Accompany the Updated Preliminary OMERACT Pediatric JAMRIS (Juvenile Idiopathic Arthritis MRI Score) Scoring System: Part II: Structural Damage Lesions. Semin. Arthritis Rheum..

[44] Bressem K.K., Adams L.C., Proft F., Hermann K.G.A., Diekhoff T., Spiller L., Niehues S.M., Makowski M.R., Hamm B., Protopopov M. (2022). Deep Learning Detects Changes Indicative of Axial Spondyloarthritis at MRI of Sacroiliac Joints. Radiology.

[45] Ożga J., Ostrogórska M., Wojciechowski W., Żuber Z. (2024). Single-Centre Analysis of Magnetic Resonance Imaging of Sacroiliac Joints in a Paediatric Population. J. Clin. Med..

[46] Carol H.A., Chauvin N.A., Weiss P.F. (2023). Imaging in Pediatric Spondyloarthritis. Curr. Opin. Rheumatol..

[47] Shenoi S., Horneff G., Aggarwal A., Ravelli A. (2024). Treatment of Non-Systemic Juvenile Idiopathic Arthritis. Nat. Rev. Rheumatol..

[48] Ravelli A., Consolaro A., Horneff G., Laxer R.M., Lovell D.J., Wulffraat N.M., Akikusa J.D., Al-Mayouf S.M., Antón J., Avcin T. (2018). Treating Juvenile Idiopathic Arthritis to Target: Recommendations of an International Task Force. Ann. Rheum. Dis..

[49] Srinivasalu H., Oliver M., Stoll M.L., Weiss P.F., Colbert R.A. (2022). Survey of Current Practices in the Management of Anti-TNF Failure in Juvenile Spondyloarthritis. Clin. Exp. Rheumatol..

[50] Burgos-Vargas R., Loyola-Sanchez A., Ramiro S., Reding-Bernal A., Alvarez-Hernandez E., van der Heijde D., Vázquez-Mellado J. (2022). A Randomized, Double-Blind, Placebo-Controlled 12-Week Trial of Infliximab in Patients with Juvenile-Onset Spondyloarthritis. Arthritis Res. Ther..

[51] Ruperto N., Foeldvari I., Alexeeva E., Ayaz N.A., Calvo I., Kasapcopur O., Chasnyk V., Hufnagel M., Żuber Z., Schulert G. (2021). Efficacy and Safety of Secukinumab in Enthesitis-Related Arthritis and Juvenile Psoriatic Arthritis: Primary Results from a Randomised, Double-Blind, Placebo Controlled, Treatment Withdrawal, Phase 3 Study. Ann. Rheum. Dis..

[52] Brunner H.I., Foeldvari I., Alexeeva E., Ayaz N.A., Calvo Penades I., Kasapcopur O., Chasnyk V.G., Hufnagel M., Zuber Z., Schulert G. (2023). Secukinumab in Enthesitis-Related Arthritis and Juvenile Psoriatic Arthritis: A Randomised, Double-Blind, Placebo-Controlled, Treatment Withdrawal, Phase 3 Trial. Ann. Rheum. Dis..

[53] Wu P., Huang C., Liao P.F., Ruperto N., Zeng H. (2023). Secukinumab in Enthesitis-Related Arthritis and Juvenile Psoriatic Arthritis. Int. J. Rheum. Dis..

[54] Baer J., Klotsche J., Foeldvari I. (2022). Secukinumab in the Treatment for Patients with Juvenile Enthesitis Related Arthritis Non-Responsive to Anti-TNF Treatment According the Juvenile Spondyloarthritis Disease Activity Index. Clin. Exp. Rheumatol..

[55] Nelson M.C., Manos C.K. (2023). Secukinumab Therapy in Refractory Juvenile Idiopathic Arthritis. J. Investig. Med. High Impact Case Rep..

[56] Marino A., De Souza M., Giani T., Cimaz R. (2020). Pharmacotherapy for Juvenile Spondyloarthritis: An Overview of the Available Therapies. Expert Opin. Pharmacother..

[57] Cohen S., Reddy V. Overview of the Janus Kinase Inhibitors for Rheumatologic and Other Inflammatory Disorders. https://www.uptodate.com/contents/overview-of-the-janus-kinase-inhibitors-for-rheumatologic-and-other-inflammatory-disorders/.

[58] Brunner H.I., Akikusa J.D., Al-Abadi E., Bohnsack J.F., Boteanu A.L., Chedeville G., Cuttica R., De La Pena W., Jung L., Kasapcopur O. (2024). Safety and Efficacy of Tofacitinib for the Treatment of Patients with Juvenile Idiopathic Arthritis: Preliminary Results of an Open-Label, Long-Term Extension Study. Ann. Rheum. Dis..

[59] Baraliakos X., van der Heijde D., Sieper J., Inman R.D., Kameda H., Li Y., Bu X., Shmagel A., Wung P., Song I.H. (2023). Efficacy and Safety of Upadacitinib in Patients with Ankylosing Spondylitis Refractory to Biologic Therapy: 1-Year Results from the Open-Label Extension of a Phase III Study. Arthritis Res. Ther..

[60] Li Y.X., Dong C., Chen B.S., Huo A.P. (2025). Therapeutic Potential of Janus Kinase Inhibitors in Juvenile Idiopathic Arthritis. Int. J. Rheum. Dis..

[61] Doeleman M.J.H., Van Maarseveen E.M., Swart J.F. (2019). Immunogenicity of Biologic Agents in Juvenile Idiopathic Arthritis: A Systematic Review and Meta-Analysis. Rheumatology.

[62] Lassoued Ferjani H., Maatallah K., Miri S., Triki W., Ben Nessib D., Kaffel D., Hamdi W. (2022). Enthesitis-Related Arthritis: Monitoring and Specific Tools. J. Pediatr..

[63] Polat M.C., Tekin Z.E., Çelikel E., Güngörer V., Kurt T., Kaplan M.M., Tekgöz N., Sezer M., Karagöl C., Coşkun S. (2023). The Juvenile Spondyloarthritis Disease Activity Index Is a Useful Tool in Enthesitis-Related Arthritis: Real-Life Data. J. Clin. Rheumatol..

[64] De Salvo S., Sacco R., Mainard N., Lucenti L., Sapienza M., Dimeglio A., Andreacchio A., Canavese F. (2024). Total Hip Arthroplasty in Patients with Common Pediatric Hip Orthopedic Pathology. J. Child. Orthop..

[65] Sequeira S.B., McCormick B.P., Hasenauer M.D., McKinstry R., Ebert F., Boucher H.R. (2024). Juvenile Idiopathic Arthritis Is Associated With Early Medical and Surgical Complications Following Primary Total Hip Arthroplasty: A National Database Study. Arthroplast. Today.

